# Risk factors for asthma and allergy associated with urban migration: background and methodology of a cross-sectional study in Afro-Ecuadorian school children in Northeastern Ecuador (Esmeraldas-SCAALA Study)

**DOI:** 10.1186/1471-2466-6-24

**Published:** 2006-09-13

**Authors:** Philip J Cooper, Martha E Chico, Maritza G Vaca, Alejandro Rodriguez, Neuza M Alcântara-Neves, Bernd Genser, Lain Pontes de Carvalho, Renato T Stein, Alvaro A Cruz, Laura C Rodrigues, Mauricio L Barreto

**Affiliations:** 1 Instituto de Microbiologia, Universidad San Francisco de Quito, Quito, Ecuador; 2 Centre for Infection, St George's University of London, London, UK; 3 Instituto de Ciências da Saúde, Universidade Federal de Bahia, Salvador, Brazil; 4 Instituto de Saúde Coletiva, Universidade Federal de Bahia, Brazil; 5 Centro de Pesquisas Gonçalo Moniz – FIOCRUZ, Salvador, Brazil; 6 Department of Pediatrics, School of Medicine, Pontifica Universidade Católica, Porto Alegre, Brazil; 7 Centro de Enfermidades Respiratórias, Faculdade de Medicina, Universidade Federal de Bahia, Salvador, Brazil; 8 London School of Hygiene and Tropical Medicine, London, UK

## Abstract

**Background:**

Asthma and allergic diseases are becoming increasingly frequent in children in urban centres of Latin America although the prevalence of allergic disease is still low in rural areas. Understanding better why the prevalence of asthma is greater in urban migrant populations and the role of risk factors such as life style and environmental exposures, may be key to understand what is behind this trend.

**Methods/design:**

The Esmeraldas-SCAALA (Social Changes, Asthma and Allergy in Latin America) study consists of cross-sectional and nested case-control studies of school children in rural and urban areas of Esmeraldas Province in Ecuador. The cross-sectional study will investigate risk factors for atopy and allergic disease in rural and migrant urban Afro-Ecuadorian school children and the nested case-control study will examine environmental, biologic and social risk factors for asthma among asthma cases and non-asthmatic controls from the cross-sectional study. Data will be collected through standardised questionnaires, skin prick testing to relevant aeroallergen extracts, stool examinations for parasites, blood sampling (for measurement of IgE, interleukins and other immunological parameters), anthropometric measurements for assessment of nutritional status, exercise testing for assessment of exercise-induced bronchospasm and dust sampling for measurement of household endotoxin and allergen levels.

**Discussion:**

The information will be used to identify the factors associated with an increased risk of asthma and allergies in migrant and urbanizing populations, to improve the understanding of the causes of the increase in asthma prevalence and to identify potentially modifiable factors to inform the design of prevention programmes to reduce the risk of allergy in urban populations in Latin America.

## Background

Large increases in the prevalence of asthma and allergic diseases have been reported in industrialized countries during the last twenty to thirty years [1,2], although there is evidence that the observed increase in asthma has reached a plateau in some industrialized countries [3,4]. There is strong evidence for differences in the prevalence of allergic diseases between urban and rural areas in Europe and in non-industrialized countries, with higher prevalences of allergic diseases reported in urban areas [5-7].

Allergic diseases are caused by a complex interaction between host genetics and environmental exposures. Temporal trends in allergy prevalence in developed countries [4], and the differences in allergy risk between urban and rural populations of the same ethnicity in developing countries [8], indicate that changes in environmental exposure rather than genetic factors are the most likely explanation for these epidemiological observations.

Rural residence is consistently identified as the strongest protective factor against asthma in epidemiological studies [7,9] and is likely to be associated with environmental exposures such as life style, diet, and hygiene [3,4,10]. Numerous epidemiological studies have provided evidence that migration from countries of low asthma prevalence (non-industrialized countries) to those of high asthma prevalence (industrialized countries) is associated with an increased risk of allergic disease [11-14], but the environmental factors that determine this have not been identified. There are few published studies of allergic diseases among migrant populations within non-industrialized countries [8] that migrate from low-risk rural areas to high-risk urban areas.

Changes in exposures to many different environmental and life style factors are likely to occur in populations that migrate from rural to urban areas, and studies of migrants to urban areas and comparisons of environmental exposures with the original rural and with more established urban populations should allow the effects of environmental exposures in early life (e.g. causing immune programming) on allergy risk to be distinguished from the effects of current exposures. Clarification of the dynamics behind the causation of allergy and asthma in urban migrant populations will further improve our understanding of what is behind the increase of frequency of these diseases with urbanization and westernization. The identification of modifiable risk factors could lead to new public health initiatives to reduce the burden of allergic disease among urbanizing populations.

Latin American countries are undergoing a rapid process of population change that includes urbanization, migration, economic development and adoption of a "modern" lifestyle. Among the burgeoning urban populations of Ecuador and other Latin American countries, asthma and allergic diseases are perceived to be an increasingly important public health problem of children [15], although there is limited data quantifying the magnitude of the problem and the associated risk factors. The International Study of Asthma and Allergies in Childhood (ISAAC) compared the prevalence of allergic disease in school-age children using a standardized questionnaire and found that urban centres in Latin America have among the highest rates of allergic symptoms worldwide [16]. School children living in rural areas of Ecuador appear to have very low risks of allergic disease [17]. Current trends of continuing rural-urban migration, coupled with the large expected increases in the urban population of Ecuador over the next 10 years, make it likely that allergic diseases, including asthma, will become a significant public health problem in the future.

The study described here is part of the Programme "Social Change, Asthma and Allergy in Latin America" (SCAALA), a research programme being conducted in Ecuador and Brazil (in the North Eastern city of Salvador), funded by The Wellcome Trust as part of the programme of Major Awards to Centres of Excellence in Latin America. The SCAALA collaboration aims to clarify the social and biological mechanisms that mediate the effect of population and lifestyle changes on the frequency of atopic diseases. This paper deals with the methodological aspects of the study being conducted in Ecuador.

The study in Ecuador aims to study changes in the prevalence and risk factors for asthma and allergy in populations that migrate from rural to urban areas and examine how such changes may relate to changes in the risk of atopic disease. The study will measure the frequency of symptoms of asthma, allergic rhinitis and atopic dermatitis in school children in urban and rural areas, and will collect detailed information on life style factors and environmental exposures that may affect the frequency of atopic diseases in the urban and rural environments. The study will investigate also changes in key immunologic factors (i.e. cytokines) and environmental exposures (i.e. exposure to aeroallergens in the household) that may be associated with altered asthma risk in the rural and urban study groups. On the other hand, the SCAALA study in Brazil aims to investigate the associations between the prevalence and incidence of allergic diseases, environmental exposures, particularly hygiene-related exposures, and immunological parameters in a cohort of children in the city of Salvador in the State of Bahia, and has been described elsewhere [18].

## Methods/design

Ecuador is among the poorest of South American countries, with an estimated per capita GDP of US$4,300 in 2005 and industrialization that is far less advanced compared to richer countries in the region, such as Chile (GDP per capita, US$11,300 and Argentina (GDP per capita, US$13,100) [19]. In Ecuador, the process of industrial development accelerated from the 1970s as a consequence of revenues available from oil exploitation in the Eastern forest region. Industrial development has been associated with a high rate of population growth in the cities, estimated to be 2.5% annually in 2000 [20]. There have been large increases in the urban proportion of Ecuador's population that rose from 42% to 65% during the period 1975 to 2000 and is projected to reach 76% by 2015 [20]. The new urban centres are home to 50% of the country's poor [20].

### Study site

The study is based in Ecuador's northern coastal province, Esmeraldas Province. The Province covers an area of 15,237 km^2 ^and has a population of approximately 429,000. The main economic activities are oil industry, tourism, timber extraction, and African palm oil. The principal city in the Province is Esmeraldas, with a population of 250,566 that is home to approximately 80% of the Ecuador's Afro-Ecuadorian population [21]. Esmeraldas Province is one of the poorest regions of Ecuador, with a per capita income that is less than half the national average. An estimated 70% of the economically active population in the city of Esmeraldas is unemployed or under-employed, and 60% have no access to basic services such as electricity, drinking water and sanitation [21]. Over the past 30 years there have been large migrations of Afro-Ecuadorians from rural areas of the Province to the city of Esmeraldas, largely as a consequence of displacement by African palm oil plantations and by migrants from other Provinces. The comparison of environmental exposures between Afro-Ecuadorians that continue to live in a rural environment with those (of the same genetic 'stock') that have migrated from the same rural environment to live in an urban environment provides an important opportunity to investigate how changes in environmental and life style factors that follow urban migration may contribute to changes in allergy risk.

### Study population

The study will be conducted among school children attending rural schools in Afro-Ecuadorian communities in the District of Eloy Alfaro, in a tropical coastal area of Esmeraldas Province, and in marginal Afro-Ecuadorian 'barrios' in an urban centre, where migrants from the rural area (called norteños) congregate.

### Study design

The study forms two parts: (i) a cross-sectional study of environmental factors associated with atopy and allergic symptoms in school-age children in the rural District of Eloy Alfaro in Esmeraldas Province and in 'barrios' of the city of Esmeraldas where rural migrants congregate; and (ii) a nested case-control study of environmental risk factors for asthma using asthma cases identified from the cross-sectional study in urban and rural school children and a random sample of non-asthmatic controls.

#### 1. Cross-sectional study

A total of 4,000 school children, aged 7 to 15 years, living in the District of Eloy Alfaro and 2,500 children of the same age range, living in the city of Esmeraldas, will be assessed to estimate the frequency of atopy and allergic diseases, including asthma, rhinitis and eczema, and identify and compare risk factors associated with these outcomes in urban and rural study populations. School children in urban areas will be defined by place of birth, period of residence in urban area and at what age, and whether their parents are migrants (i.e. born in a rural area) or not. The cross-sectional study will examine the relationship between atopy and allergic symptoms in urban and rural school children and the environmental factors that modify this relationship.

#### 2. Nested case-control study

Asthma cases (200 cases in each of the urban and rural areas) and non-asthmatic controls (800 controls in each of the rural and urban areas), respectively, will be identified from the results of an allergy symptom questionnaire performed in the cross-sectional study by the questions – "Have you had wheezing or whistling in the chest in the last 12 months? " and "Have you ever had wheezing or whistling in the chest at any time in the past?" Non-asthmatic controls will be randomly selected from all children in each of the urban and rural areas that respond negatively to the second question. Nested case-control studies in the urban and rural areas will identify risk factors associated with symptoms of recent wheeze using both quantitative and qualitative epidemiological methods, investigations of immunological function and measurements of allergens and endotoxin in the environment.

### Sample size and study power

Data are available for the prevalence of recent wheeze in urban and rural study areas, from pilot surveys of 245 and 536 school children, respectively. These surveys estimate the prevalence of recent wheeze to be greater in the urban area (urban 13.4% versus rural 7.3%). The prevalence of atopy was similar in urban and rural school children (urban 11.3% vs. rural 13.5%). A sample of 4,000 children in the rural area and 2,500 children in the urban area will provide approximately 820 cases of atopy for the cross-sectional study, and 292 and 335 cases of asthma for the case-control study in the rural and urban areas, respectively. Two hundred asthma cases from each area will be recruited into case-control studies (allowing a drop-out rate of ~ 30%). The study will have a case-control ratio of 1:4 (400 cases vs. 1,600 controls). With a study power of 80% and P < 0.05, the nested case-control studies combined (i.e. urban and rural) will be able to detect significant effects on asthma of common exposures (prevalences of 40–60% – e.g. geohelminth infections and household pets) with OR<0.7 and rare exposures (10%, e.g. family history of allergic disease) with OR<0.5. Likewise, the individual case-control studies (i.e. urban vs. rural) with the same power and level of significance will be able to detect an effect of OR<0.6 for common exposures (40–60% prevalence), and OR<0.4 for rare exposures (10%).

### Main exposures and definitions

Asthma, rhinitis and eczema will be defined according to the core allergy symptom questions of the International Study of Allergy and Asthma in Childhood (ISAAC) [22]. Atopy will be defined as a positive skin prick test to any of the panel of aeroallergens tested. Allergy will be defined by the presence of a history of appropriate symptoms of a clinical allergic condition (asthma, rhinitis or eczema) in the presence of atopy [23]. Information on important risk factors and environmental exposures that affect the risk of atopy and asthma will be collected as listed in Table 1.

### Data collection and instruments

#### 1. Questionnaires

*A. Cross-sectional study*: The questionnaire has been modified from the ISAAC Phase II study questionnaire [24], and has been translated into Spanish and extensively field tested [17,25]). The questionnaire includes the core allergy symptoms questions of ISAAC and will collect information on maternal and paternal educational level and occupation; house construction; family possession of material goods; family income; cooking materials; access to water, electricity, and sanitation; household crowding; exposures to pets and animals; exposures to farming, including consumption of unpasteurized milk; dietary questionnaire; physical activity and time spent watching television; number of siblings and birth order; breast feeding; day care attendance; family history of allergic disease; exposure to smoking; detailed history of residence in urban and rural areas of father and mother; and detailed history of residence in urban and rural areas of child. The questionnaire will be administered to the parent or guardian of each child by trained field workers in the presence of the child.

*B. Case-control study*: A questionnaire for a more detailed history of asthma, rhinitis and eczema symptoms, based on the ISAAC phase II supplementary questionnaires [24], will be used. The questionnaire is designed to distinguish between asthma and other common respiratory disorders. The questionnaire includes also questions of the management of asthma [24] and of risk factors for asthma. The case-control study will include qualitative epidemiological techniques to assess knowledge, attitudes, and practice about asthma (KAP questionnaire). KAP questionnaires will be administered to the parents of asthma cases and non-asthmatic controls and also to health care personnel in health centres in urban and rural areas. Deep interviews with key informants in urban barrios and rural communities, focus groups and other qualitative epidemiological techniques will be used to evaluate relevant lifestyle factors, and how different patterns between urban and rural areas may contribute to risk (e.g. factors that are protective in rural areas, such as rearing of livestock, may be risk factors in urban areas as recent migrants 'ruralise' their urban environment).

#### 2. Exercise test

The presence of exercise-induced bronchospasm (EIB) will be assessed in all children in the case-control as described previously [25,26]. Briefly, peak expiratory flow rate (PEFR) is measured before and after 6 minutes of vigorous exercise and a fall of 15% in PEFR is considered to indicate EIB.

#### 2. Nutritional assessment

Finger prick blood samples will be collected from all subjects in the cross-sectional study to estimate hemoglobin level using standard procedures. Anthropometric measurements will be performed using standardised methodology and will include weight (kg), height (cm) and triceps skin fold thickness (mm). *z*-scores for weight-for-age, weight-for-height and height-for-age will be calculated using the EPINUT program (Epi Info 6.0; CDC. Atlanta, GA, USA).

#### 3. Examination of stool samples for parasites

Single stool samples will be collected from all children and examined using the modified Kato-Katz and formol-ether concentration methods [27]. Intestinal helminth parasite burdens (Kato-Katz) will be quantified as eggs per gram of faeces. Infections with protozoan pathogens (formol-ether concentration) will be graded as positive or negative. All children with intestinal helminth infections will be offered appropriate treatments with albendazole and children with trophozoites of *Entamoeba histolytica *(with ingested red cells) and *Giardia intestinalis *will be offered appropriate doses of tinidazole.

#### 4. Examination of dust samples

Dust samples will be collected from the homes of the children in the case-control study using a ~ 1200 W vacuum cleaner, weighed and stored as described previously [18]. Surveys of mites in dust samples and of cockroaches in houses in the rural area show a predominance (>90%) of both *Dermatophagoides pteronyssinus *mite and the American cockroach, *Periplaneta americana*. Dust samples will be analyzed for endotoxin and fungal β-glucans (Limulus lysate assay, BioWhittaker, MD, USA) and allergens from *D. pteronyssinus *(Der p 1) and *P. americana *(Per a 1) (Indoor Biotechnologies, VA, USA).

#### 5. Examination of blood samples

Blood samples (7 mL) will be collected by venipuncture from participants in the case-control study and will be used for obtaining serum for the measurement of: polyclonal IgE [28]; IgE antibodies specific for *D. pteronyssinus*, American cockroach, and *Ascaris lumbricoides *using the Pharmacia CAP system (Phadia AB, Uppsala, Sweden); IgG antibodies specific for hepatitis A virus, *Helicobacter pylori*, herpes simplex virus, herpes zoster virus, Epstein-Barr virus, and *Toxoplasma gondii *using commercially available assays; and for whole blood cultures stimulated with mitogen and relevant allergens for the measurement of the regulatory cytokines, IFN-γ, IL-13, and IL-10, as described elsewhere [18]. A whole blood pellet sample will be stored at -40°C and shipped frozen to Imperial College, London, UK, for genotyping of single nucleotide polymorphisms using the Sequenom system (Sequenom Inc, San Diego, CA, USA), as described previously [24].

#### 6. Allergen skin prick testing

All children in the cross-sectional study will be tested for immediate hypersensitivity responses to relevant aeroallergens as described previously [25,28]. A positive test will be taken as a wheal with a mean diameter of at least 3 mm greater than the saline control 15 minutes after pricking the allergen into the right forearm using an ALK Lancet (ALK-Abello, Horsholm, Denmark). The following allergens from Greer Laboratories Inc (Lenoir, NC, USA) will be tested: *D. pteronyssinus/D. farinae *mix, American cockroach, *Alternaria tenuis*, cat, dog, '9 Southern grass mix', and 'New stock fungi mix' .

### Overview of statistical plan of analysis

The analysis will be designed to address five principal study questions: 1) What is the frequency of atopy and asthma in school children from the rural and urban study areas? 2) What environmental exposures are associated with atopy and asthma and how do these interact with area of residence (rural vs. urban) to affect the relationship between atopy and wheeze symptoms? 3) Do environmental exposures associated with wheeze differ between migrant and established urban populations and, if so, are they potentially modifiable through intervention programmes? 4) Are place of birth (rural vs. urban) and/or period of residence in rural and urban areas associated with risk of allergy? 5) How do complex inter-relationships between factors at different levels (e.g. immunologic factors and social factors) interact to affect the risk of asthma in urban and rural populations? Statistical analysis will be conducted according to a conceptual framework that defines a proposed causal pathway and the complex analytic approach to be used has been described in detail in a companion paper [18]. Methods (epidemiological and laboratory measurements) have been standardized between the Salvador-SCAALA [18] and this study (Esmeraldas-SCAALA), a procedure that will permit comparisons between the two studies, and may allow the identification of common risk/protective factors as well as those that are peculiar to the different study sites.

### Ethical considerations

Ethical approval for the study has been obtained from the Hospital Pedro Vicente Maldonado, Provincia de Pichincha, Ecuador. Written informed consent to participate in the study will be obtained from the parent of each child and signed minor assent will be obtained from each child. The parent or guardian of each child will be provided with a copy of all laboratory results and if, appropriate, treatment recommendations will be made by a trained clinician that will review each case.

## Discussion

Atopic asthma and other allergic diseases are becoming increasingly important public health problems in Latin American cities and there is little published information on the causes of this disease epidemic. The SCAALA (Social Change, Asthma and Allergy in Latin America) research initiative includes two epidemiological studies being conducted in urbanizing populations (Esmeraldas Province, Ecuador and the city of Salvador in Brazil, respectively) that are investigating the environmental causes of allergy in urban Latin America and the biological and social mechanisms that underlie these epidemiological trends. While the SCAALA-Salvador aims to investigate the associations between the prevalence of asthma and other allergic diseases (rhinitis, atopic eczema) and potential risk factors that includes living conditions and early life and current exposures to infections [18], the SCAALA-Esmeraldas study aims to study frequency of atopy and allergic diseases and exposure to potential risk factor in rural populations and in migrants from rural to urban areas and examine how these may explain the risk of atopic diseases in migrants from rural to urban areas. Both studies will investigate how the association between environment factors, allergic diseases and markers of atopy (i.e. skin-prick test and total and specific serum IgE levels) may be mediated by interleukins from antigen-stimulated leukocytes.

The Esmeraldas-SCAALA study in Ecuador includes cross-sectional and nested case-control studies conducted in rural and urban contexts. The studies are investigating the impact of urban migration on asthma risk and the environmental exposures that are associated with an increased risk of asthma in populations that migrate from rural to urban areas. The study will focus on a single ethnic cultural group, Afro-Ecuadorians, that traditionally has lived in the remote rural North Eastern region of Esmeraldas Province, but that has migrated in significant numbers over the past 30 years to cities such as the provincial capital, Esmeraldas. The study of a single and easily identifiable group that presumably shares the same genetic 'stock' and that has migrated locally (within the same Province) should allow important environmental risk factors to be identified more easily and should not suffer from the biases that limit the interpretation of studies that have investigated populations that have migrated between countries. Specifically, the study, by investigating a migrant population, will distinguish between the effects of early life exposures (inducing immune programming) and current exposures in determining allergy risk.

The knowledge generated from this study will help to define the size of the public health problem of allergy in Ecuador and may identify possible environmental exposures that could be considered for primary prevention public health strategies. The study in Brazil is a prospective study investigating the effects of early life exposures to environmental factors, and the potential effects of these on the immune system and the risk of allergy and has been described in detail in a separate paper [18].

The causes of the allergy epidemic in Latin America are assumed to be multifactorial and an important strength of the SCAALA studies is the use of similar causal frameworks and the sharing of methodology and expertise in a wide range of scientific disciplines (e.g. epidemiology, immunology, microbiology, biostatistics and social sciences). The two studies are complementary and are likely to yield important information on the underlying causes of the allergy epidemic in urban Latin America.

In summary, the aim of the proposed programme is to investigate the biological and social mechanisms that underlie the epidemic of allergic diseases in urban Latin America. Urbanization and the health problems associated with this phenomenon probably represent the single most important challenge for health researchers working in developing countries in the 21^st ^century, and allergic diseases are likely to emerge as the most prevalent of chronic diseases of childhood in Latin America during this century. Latin America urbanization has its roots on the intense process of displacement of the poor rural population that move to the urban centres looking for work and other improvements in their life conditions. It is expected that the knowledge generated from the SCAALA studies will help identify public health interventions that may ameliorate the adverse effects of urbanization on the prevalence and severity of asthma and allergic diseases.

## Abbreviations

ISAAC: International Study of Asthma and Allergies in Childhood

SCAALA: Social Change, Asthma and Allergy in Latin America

## Competing interests

The author(s) declare that they have no competing interests.

## Authors' contributions

PJC and LCR had the original idea for the study. PJC designed the study and drafted the manuscript. MC, MV were involved in study design and co-ordination. NAN and LPC were responsible for the immunological methods. BG was responsible for the statistical analysis plan. LR, AR, AC, RS and MB were involved in study design. All authors helped draft the manuscript, and read and approved the final version of the manuscript.

## Pre-publication history

The pre-publication history for this paper can be accessed here:



## Figures and Tables

**Table 1:** Variables to be collected in the cross-sectional and nested case-control studies

	Cross-sectional	Case-control
**Allergy questions**		
Core asthma symptom questions (ISAAC phase II)	X	X
Core rhinitis symptom questions (ISAAC phase II)	X	X
Core eczema symptom questions (ISAAC phase II)	X	X
Supplementary asthma questions (ISAAC phase II)		X
Asthma management questions (ISAAC phase II)		X
Rhinitis management questions (ISAAC phase II)		X
Eczema management questions (ISAAC phase II)		X
Knowledge attitude practice questions (KAP)		X
		
**Demographic and socio-economic**		
Sex	X	X
Age	X	X
Race	X	X
On socio-economic level:		
parental schooling	X	
parental occupation	X	X
domestic goods	X	X
monthly income	X	X
		
**Environmental factors**		
Sanitation and water supply	X	X
Does the house have electricity?	X	X
Pets		
Has the child ever had a cat or dog living in the house?	X	
Does the child have a cat or dog living in the house currently?	X	X
Presence of animals outside household	X	X
Indoor smoking	X	X
Exposure to a farming environment	X	X
Housing (e.g. construction, number of rooms)	X	X
Presence of allergens in dust (mites and cockroach)		X
Presence of endotoxin in the house		X
Cooking materials	X	X
		
**Maternal and family related factors**		
Maternal smoking during pregnancy	X	
Asthma and other allergic diseases in the family	X	
Breast-feeding duration	X	
Vaccination (BCG, DTP, measles)	X	
Whether attended day care (and for what period)	X	
Number of people who live in each household	X	X
Number of siblings or children in the household	X	X
Birth order	X	X
		
**Diet**		
Food frequency questionnaire	X	X
Consumption of unpasteurized milk	X	
		
**Migration**		
Place of birth of child	X	
Place of birth of father	X	
Place of birth of mother	X	
Was the father brought up in an urban or rural area?	X	
Was the mother brought up in an urban or rural area?	X	
Period of time and places living outside place of birth during first 5 years of life	X	
Details of relatives living in the city of Esmeraldas	X	
		
**Exercise/sedentarism**		
Number of hours of television watched daily	X	X
Frequency of vigorous exercise	X	X
		
**Nutritional assessment**		
Hemoglobin	X	X
Height	X	X
Weight	X	X
Mid-upper arm circumference	X	
Triceps skin fold thickness	X	
		
**Specific Infections**		
Intestinal helminth and protozoal infections (stool examinations)	X	X
*A. lumbricoides *IgE		X
IgG for *Toxoplasma. Gondii*, Helicobacter pylori and viral infections		X
		
**Exercise test for exercise-induced bronchospasm**		X
		
**Atopy**		
Skin prick test	X	X
Total and specific IgE titres to house dust mite and American cockroach		X
		
**Immunoregulatory cytokines**		
IL-10, IL-13, IFN-γ		X
